# Supplementary material for: Structural insights into the regulation of Cas7-11 by TPR-CHAT
Source: Nat Struct Mol Biol. 2022 Dec 5;30(2):135–9. doi: 10.1038/s41594-022-00894-5 (PMC9935389; doi:10.1038/s41594-022-00894-5)
Supplement: Supplementary file 1 — Supplementary Figs. 1–4 [file 41594_2022_894_MOESM1_ESM.pdf]

nature structural & molecular biology

Brief Communication https://doi.org/10.1038/s41594-022-00894-5

# Structural insights into the regulation of Cas7-11 by TPR-CHAT

In the format provided by the authors and unedited

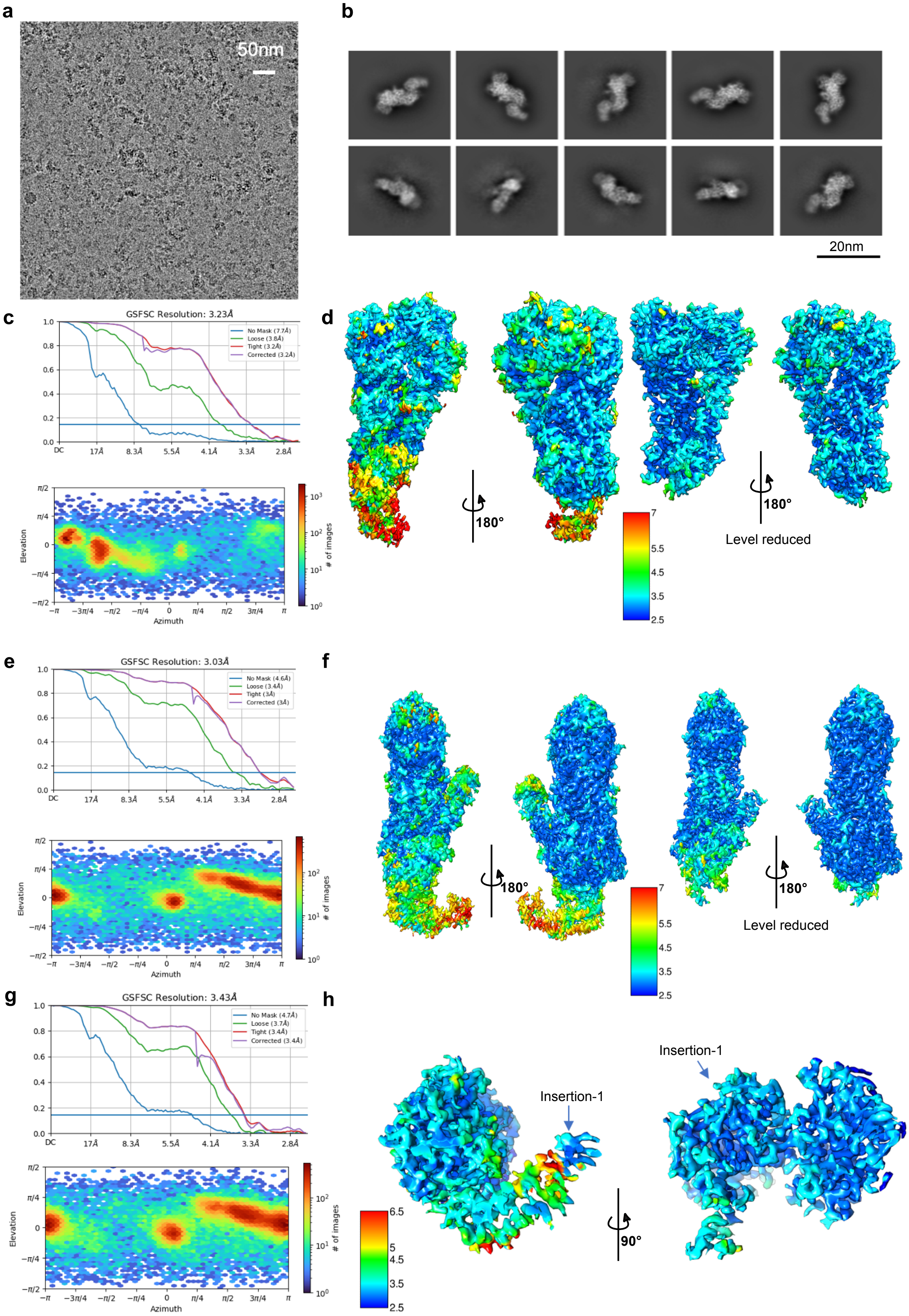
a
50nm
b
20nm
c
GSFSC Resolution: 3.23Å
No Mask (7.7Å)
Loose (3.8Å)
Tight (3.2Å)
Corrected (3.2Å)
DC
17Å
8.3Å
5.5Å
4.1Å
3.3Å
2.8Å
Elevation
Azimuth
# of images
d
180°
180°
Level reduced
7
5.5
4.5
3.5
2.5
e
GSFSC Resolution: 3.03Å
No Mask (4.6Å)
Loose (3.4Å)
Tight (3Å)
Corrected (3Å)
Elevation
Azimuth
# of images
f
180°
180°
Level reduced
7
5.5
4.5
3.5
2.5
g
GSFSC Resolution: 3.43Å
No Mask (4.7Å)
Loose (3.7Å)
Tight (3.4Å)
Corrected (3.4Å)
Elevation
Azimuth
# of images
h
Insertion-1
Insertion-1
90°
6.5
5
4.5
3.5
2.5

v

**Supplementary Figure 1: CryoEM 3D reconstruction.** a) A raw micrograph of the Craspase complex (n=18,599). b) 2D classes of the Craspase complex. c and d) The FSC curve, viewing direction distribution, and local resolution map DmCas7-11-crRNA and *Dm*TPR-CHAT$_{full}$. e and f) The FSC curve, viewing direction distribution, and local resolution map DmCas7-11-crRNA and *Dm*TPR-CHAT$_{NTD}$. g and h) The FSC curve, viewing direction distribution, and local resolution map of Cas7.4 Insertion-1 and -2 subdomains.

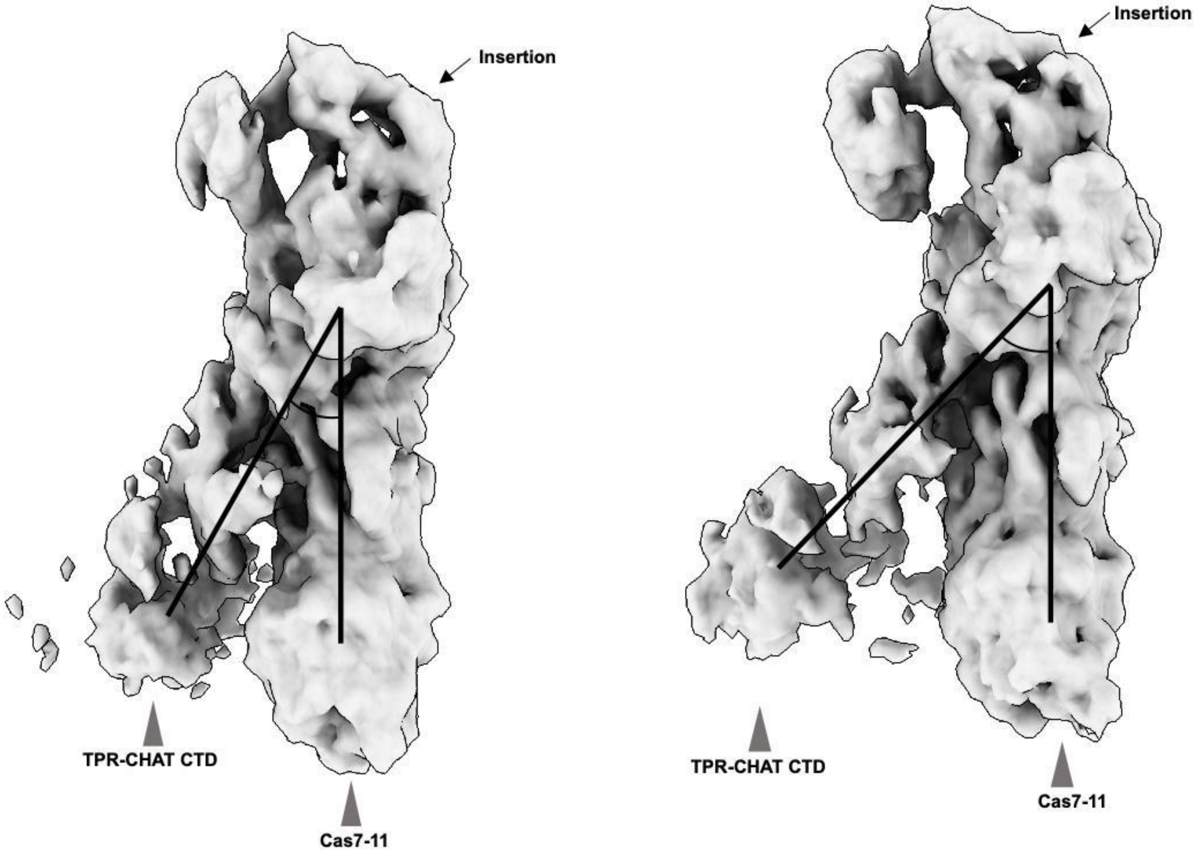


**Supplementary Figure 2: Movements of *Dm*TPR-CHAT and Insertion subdomain observed in cryo-EM maps.**

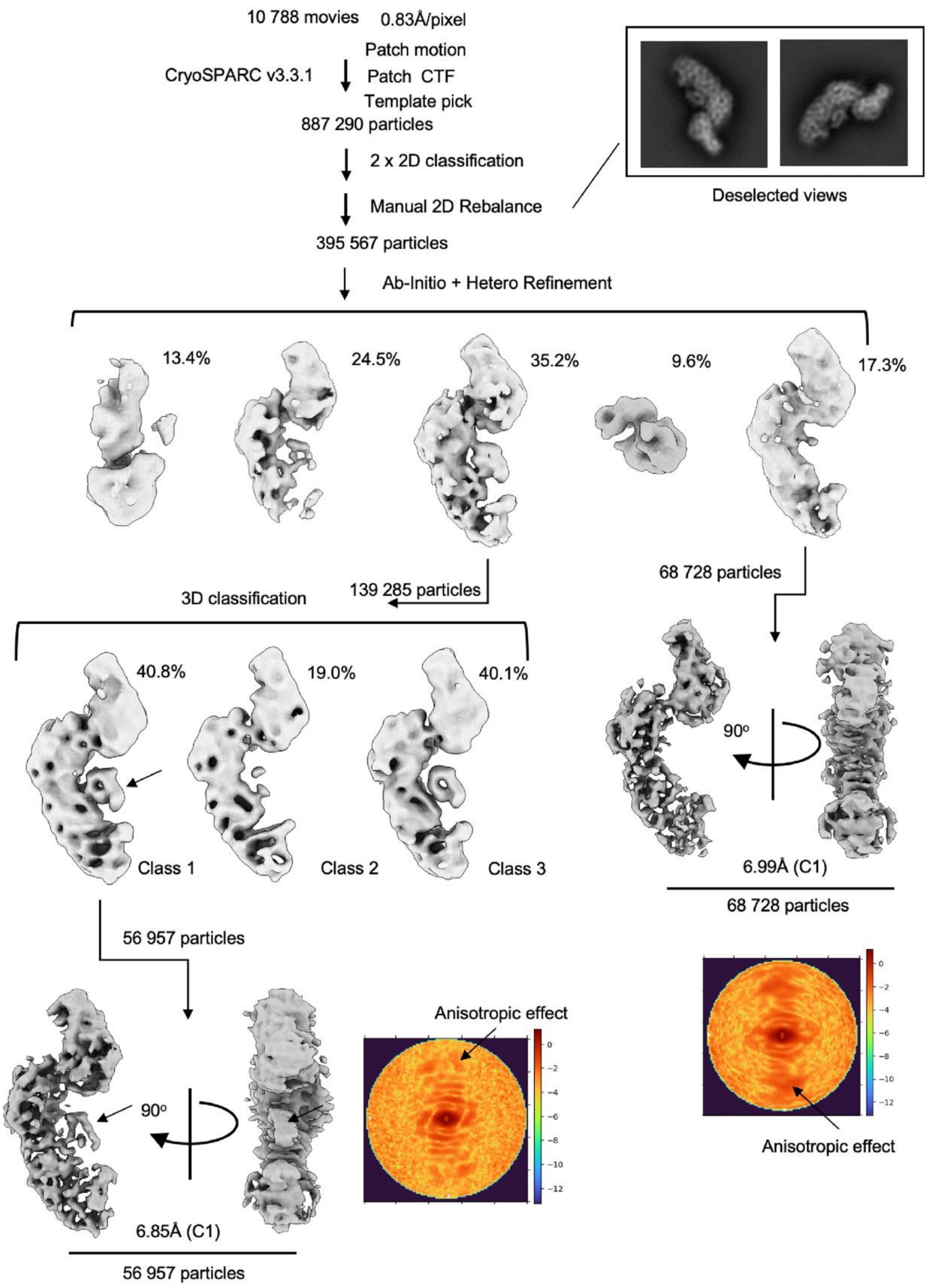


**Supplementary Figure 3: Cryo-EM data processing workflow for *D. magnum* Cas7-11-crRNA**

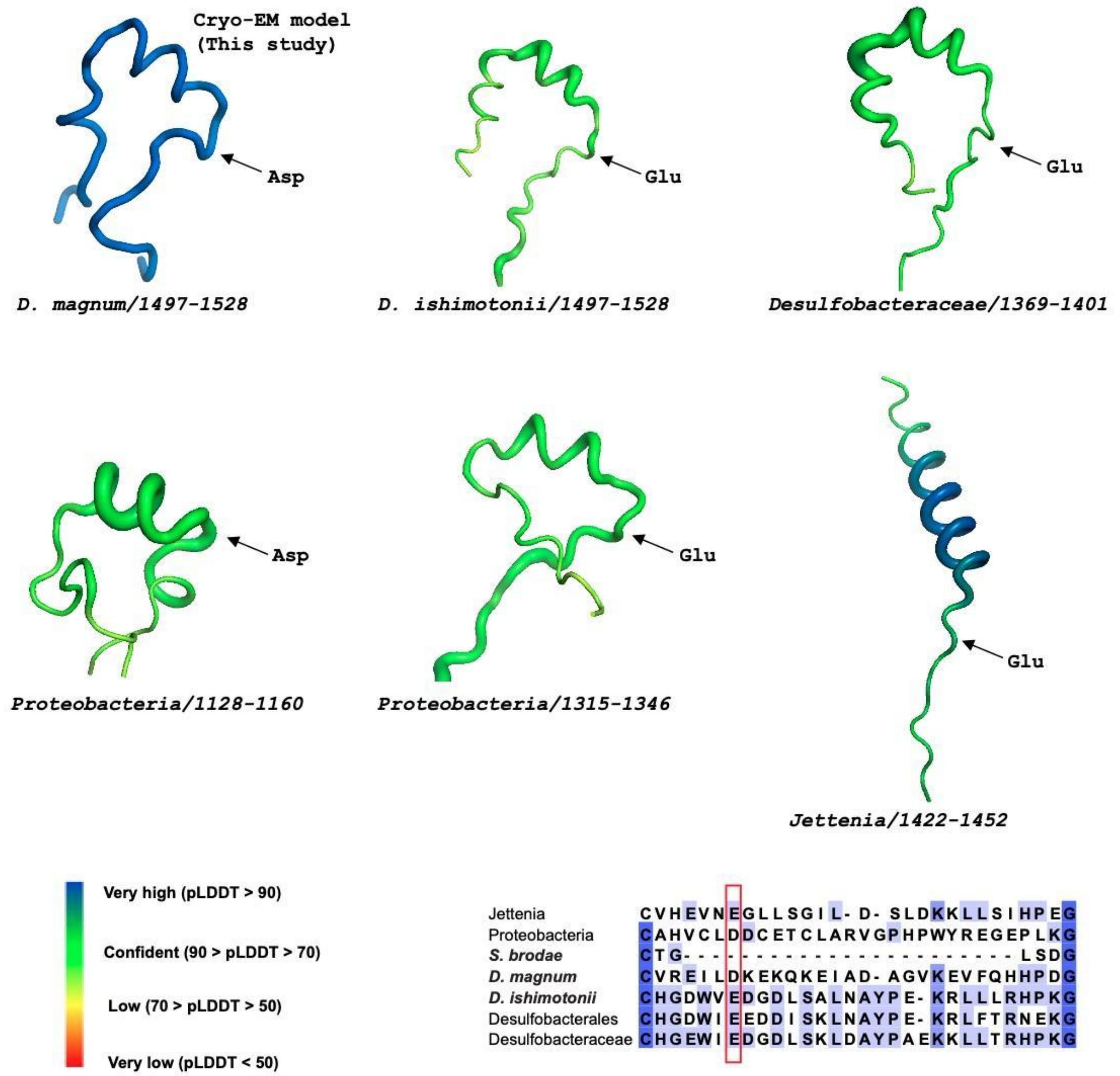


**Supplementary Figure 4: Alpha fold models of the Insertion-finger of Cas7-11 from different species**
