# Supplementary material for: Structural insights into the regulation of Cas7-11 by TPR-CHAT
Source: Nat Struct Mol Biol. 2022 Dec 5;30(2):135–9. doi: 10.1038/s41594-022-00894-5 (PMC9935389; doi:10.1038/s41594-022-00894-5)
Supplement: Supplementary file 2 — Reporting Summary [file 41594_2022_894_MOESM2_ESM.pdf]

nature portfolio

Corresponding author(s): Henning Stahlberg

Last updated by author(s): Oct 19, 2022

Reporting Summary

Nature Portfolio wishes to improve the reproducibility of the work that we publish. This form provides structure for consistency and transparency in reporting. For further information on Nature Portfolio policies, see our Editorial Policies and the Editorial Policy Checklist.

Statistics

For all statistical analyses, confirm that the following items are present in the figure legend, table legend, main text, or Methods section.

n/a	Confirmed	
☐	☒	The exact sample size (n) for each experimental group/condition, given as a discrete number and unit of measurement
☐	☒	A statement on whether measurements were taken from distinct samples or whether the same sample was measured repeatedly
☒	☐	The statistical test(s) used AND whether they are one- or two-sided<br>*Only common tests should be described solely by name; describe more complex techniques in the Methods section.*
☒	☐	A description of all covariates tested
☒	☐	A description of any assumptions or corrections, such as tests of normality and adjustment for multiple comparisons
☒	☐	A full description of the statistical parameters including central tendency (e.g. means) or other basic estimates (e.g. regression coefficient) AND variation (e.g. standard deviation) or associated estimates of uncertainty (e.g. confidence intervals)
☒	☐	For null hypothesis testing, the test statistic (e.g. F, t, r) with confidence intervals, effect sizes, degrees of freedom and P value noted<br>*Give P values as exact values whenever suitable.*
☒	☐	For Bayesian analysis, information on the choice of priors and Markov chain Monte Carlo settings
☒	☐	For hierarchical and complex designs, identification of the appropriate level for tests and full reporting of outcomes
☒	☐	Estimates of effect sizes (e.g. Cohen's d, Pearson's r), indicating how they were calculated

Our web collection on statistics for biologists contains articles on many of the points above.

Software and code

Policy information about availability of computer code

Data collection	EPU (Thermo Fisher Scientific), an iBright FL1500 Imaging System (Thermo Fisher Scientific), Unicorn V7.1
Data analysis	CryoSPARC v3.3, Coot v 0.9.4 , Phenix v 1.19.2-4158 USCF Chimera , USCF Chimera X, Pymolv1.8.2.0, Adobe Illustrator, Geneious prime (v2022.2), ImageJ (v1.53k), Clustal Omega, Jalview

For manuscripts utilizing custom algorithms or software that are central to the research but not yet described in published literature, software must be made available to editors and reviewers. We strongly encourage code deposition in a community repository (e.g. GitHub). See the Nature Portfolio guidelines for submitting code & software for further information.

Data

Policy information about availability of data

All manuscripts must include a data availability statement. This statement should provide the following information, where applicable:

- Accession codes, unique identifiers, or web links for publicly available datasets
- A description of any restrictions on data availability
- For clinical datasets or third party data, please ensure that the statement adheres to our policy

The raw image data are available from the EMPIAR database under access codes EMPIAR-11268. The reconstructed maps are available from the EMDB database under access codes EMDB-14847 and EMDB-14848. The atomic models are available in the PDB database, access codes PDB-ID 7ZOL and 7ZOQ.

Human research participants

Policy information about studies involving human research participants and Sex and Gender in Research.

Reporting on sex and gender	n/a
Population characteristics	n/a
Recruitment	n/a
Ethics oversight	n/a

Note that full information on the approval of the study protocol must also be provided in the manuscript.

Field-specific reporting

Please select the one below that is the best fit for your research. If you are not sure, read the appropriate sections before making your selection.

☒ Life sciences ☐ Behavioural & social sciences ☐ Ecological, evolutionary & environmental sciences

For a reference copy of the document with all sections, see nature.com/documents/nr-reporting-summary-flat.pdf

Life sciences study design

All studies must disclose on these points even when the disclosure is negative.

Sample size	Sample size for Cryo-EM data was determined by collecting data in the form of movies containing particle images to obtain a sufficient number of particles images that would provide high resolution 3D reconstruction following refinement based on the FSC threshold of 0.143. For the data of the Craspase (DmCas7-11-TPR-CHAT) complex, 18559 movies were collected. 65'755 particles was refined and yielded a cryo-EM map at 3.20 Å overall resolution and another set of 53'969 particles produced a cryo-EM map at an overall resolution of 3.03 Å based on FSC threshold of 0.143. Therefore, the number of particles obtained were sufficient to get high resolution structures sufficient to make the interpretations detailed in the manuscript.<br>For other experiments, no sample-size determination was performed, as the biochemical experiments followed standard practices. Replicates of all biochemical experiments was performed in order to ensure reproducibility of the results.
Data exclusions	Images suffering image drift, ice contamination, and/or cubic ice format were excluded during image processing. Particles in 2D classes showing no secondary structural features and in 3D classes showing unsatisfactory structural features were excluded from the final reconstructions in all datasets analyzed.
Replication	Cryo-EM data was obtained from 18559 observations in the form of movies from a single sample from a round of data collection. Biochemical asssays such as the nuclease activity assays were performed at least three independent times with similar results. EMSA assay was independently duplicated also with similar results.
Randomization	No randomization was involved as this study did not involve use of sample collection from different experimental groups involving participants
Blinding	Blinding is not applicable as this study did not involve sampling different experimental groups involving participants

Reporting for specific materials, systems and methods

We require information from authors about some types of materials, experimental systems and methods used in many studies. Here, indicate whether each material, system or method listed is relevant to your study. If you are not sure if a list item applies to your research, read the appropriate section before selecting a response.

Materials & experimental systems

n/a	Involved in the study	
☒	☐	Antibodies
☒	☐	Eukaryotic cell lines
☒	☐	Palaeontology and archaeology
☒	☐	Animals and other organisms
☒	☐	Clinical data
☒	☐	Dual use research of concern

Methods

n/a	Involved in the study	
☒	☐	ChIP-seq
☒	☐	Flow cytometry
☒	☐	MRI-based neuroimaging
