# Supplementary figures and images for: Structural insights into the regulation of Cas7-11 by TPR-CHAT
Source: Nat Struct Mol Biol. 2022 Dec 5;30(2):135–9. doi: 10.1038/s41594-022-00894-5 (PMC9935389; doi:10.1038/s41594-022-00894-5)

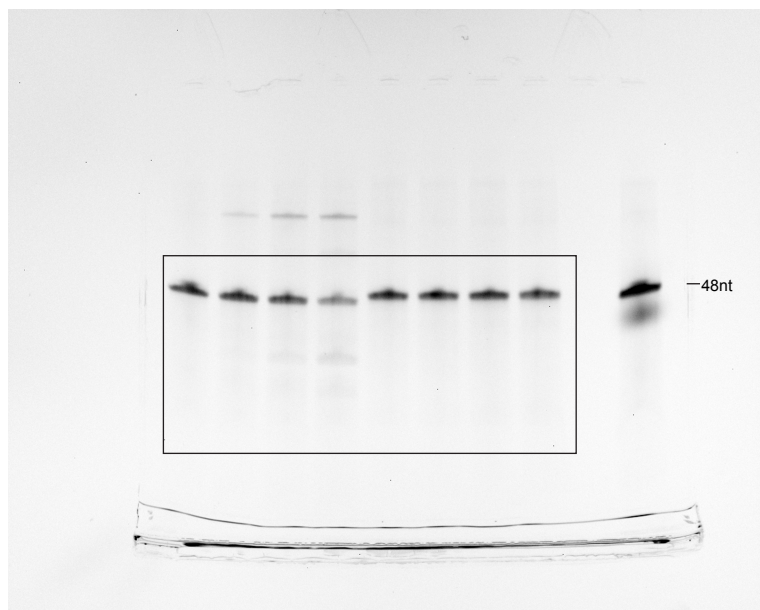

Supplement: Source Data Fig. 1 — Unprocessed gels. [file 41594_2022_894_MOESM8_ESM.pdf]

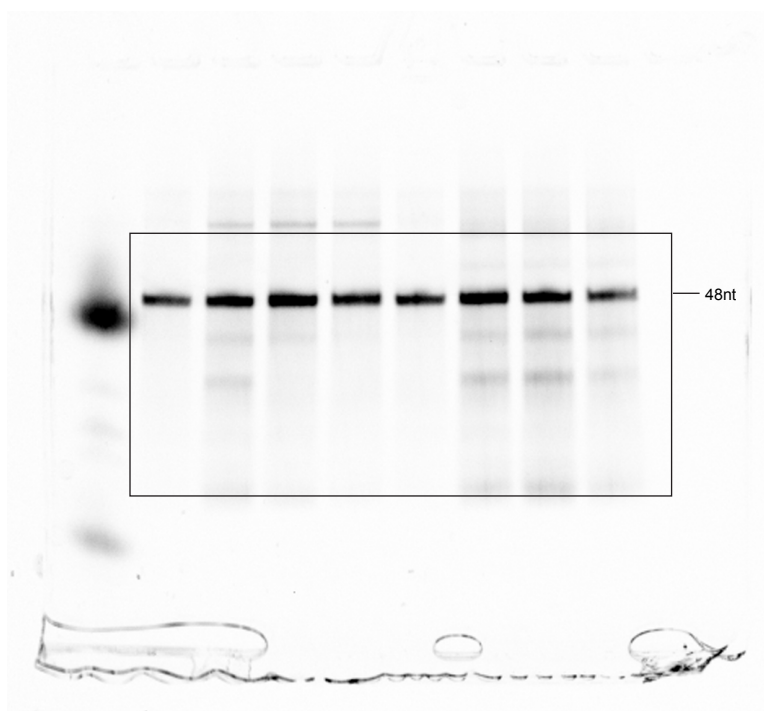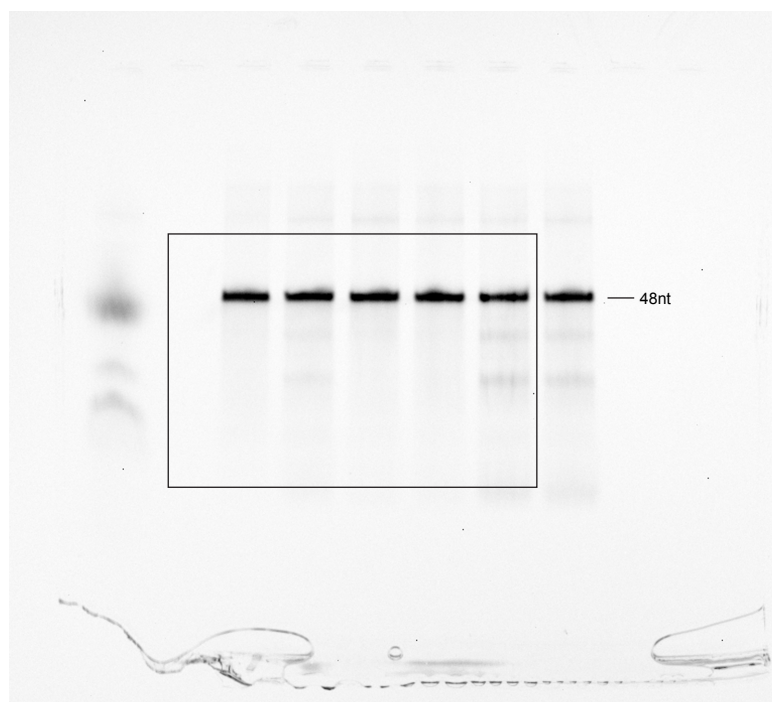

Supplement: Source Data Fig. 2 — Unprocessed gels. [file 41594_2022_894_MOESM9_ESM.pdf]

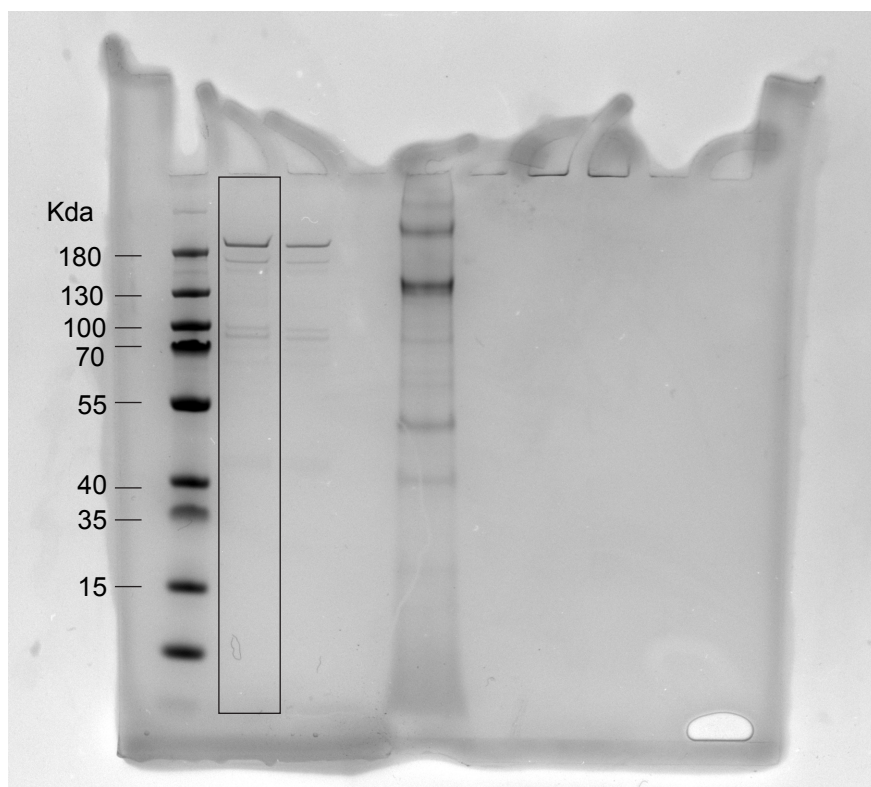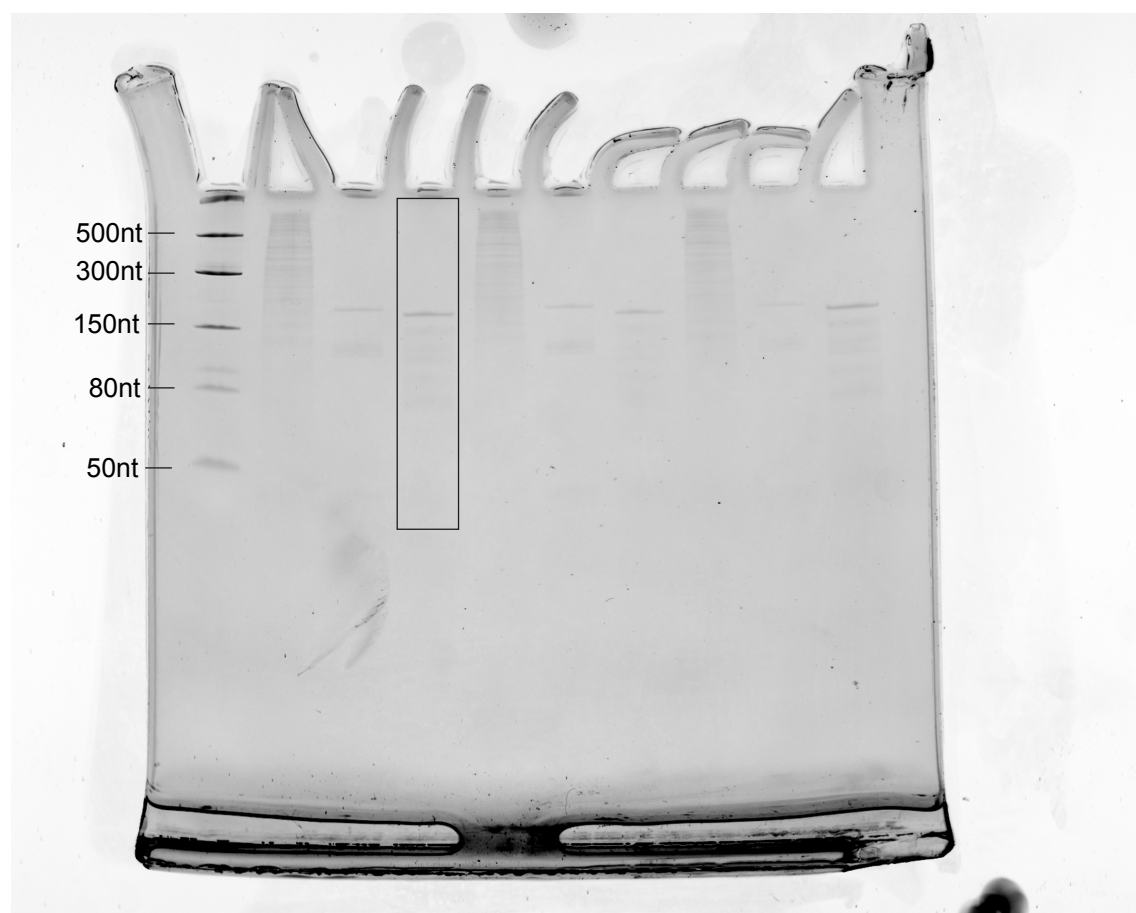

Supplement: Source Data Extended Data Fig. 1 — Unprocessed gels. [file 41594_2022_894_MOESM10_ESM.pdf]

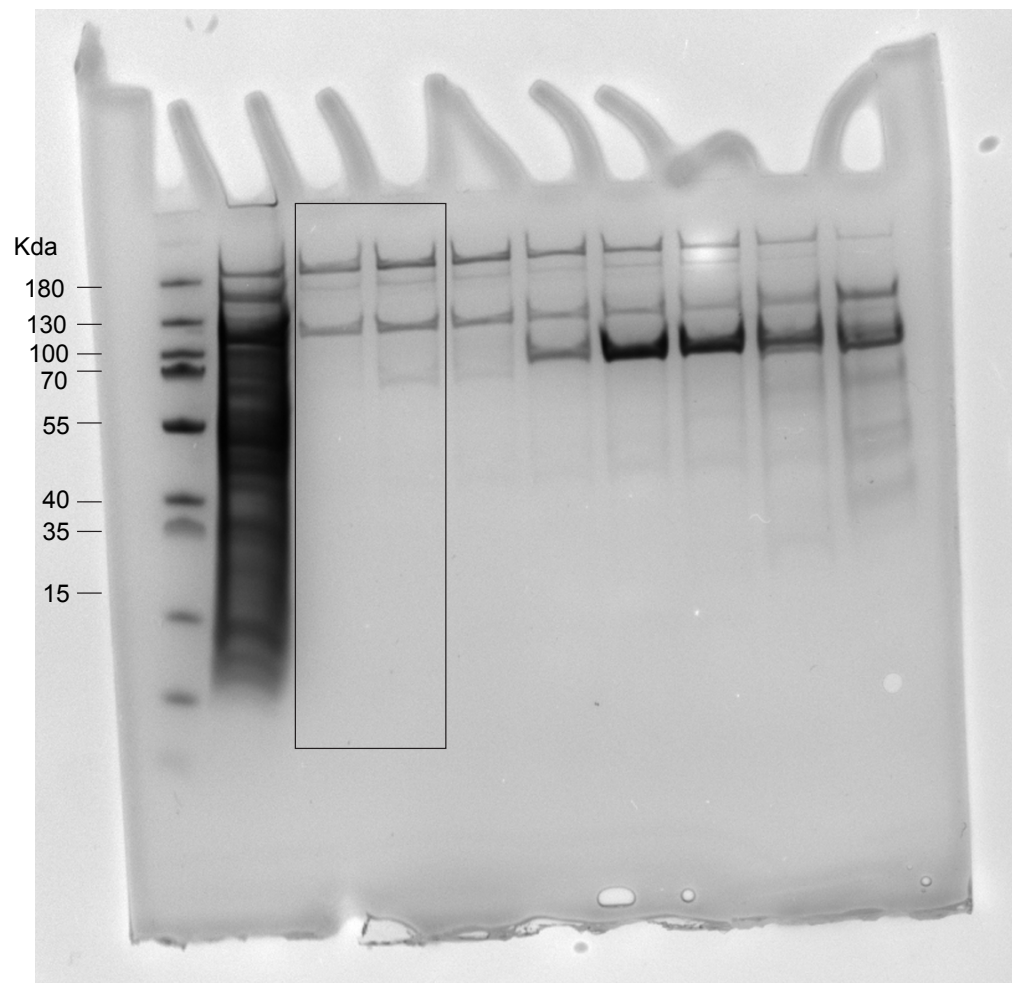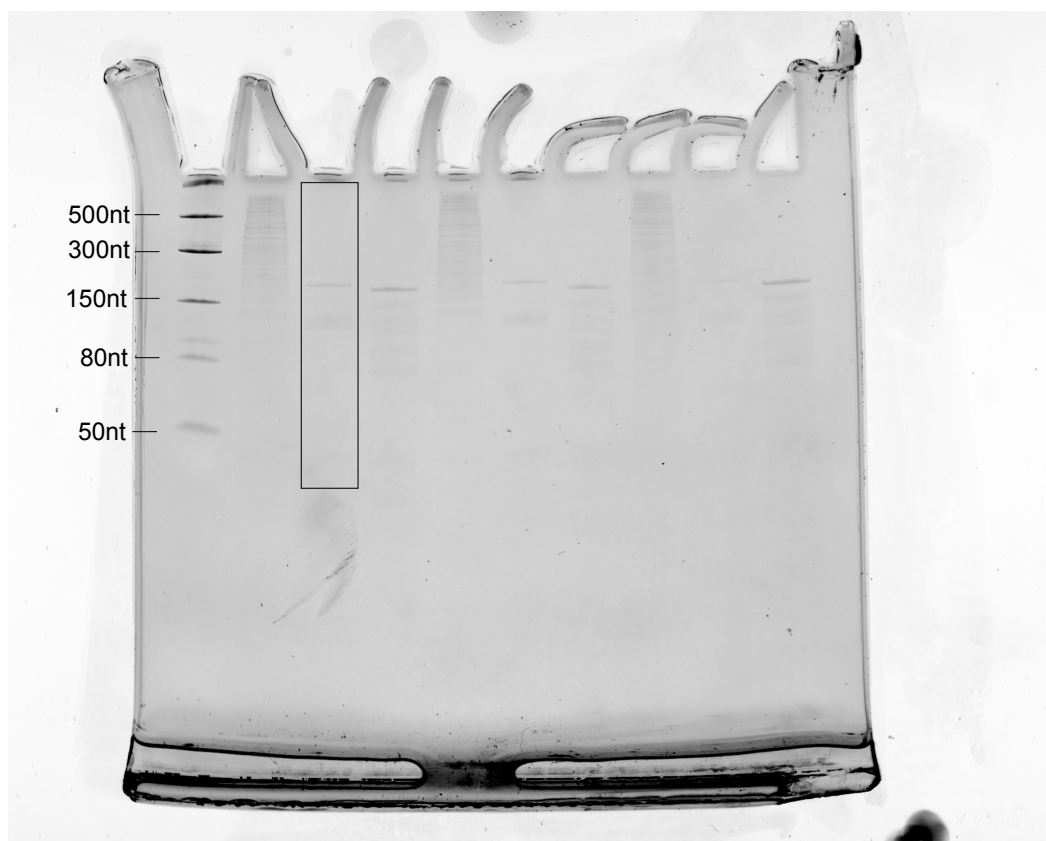

Supplement: Source Data Extended Data Fig. 2 — Unprocessed gels. [file 41594_2022_894_MOESM11_ESM.pdf]

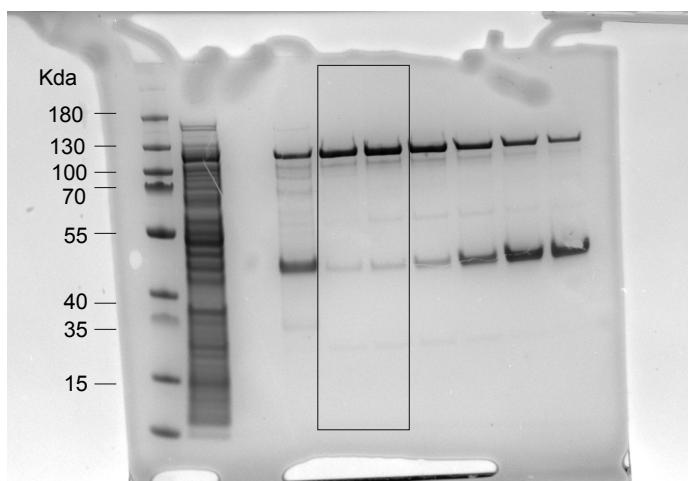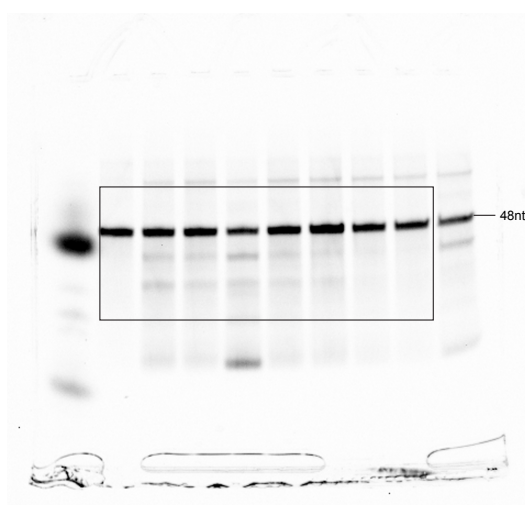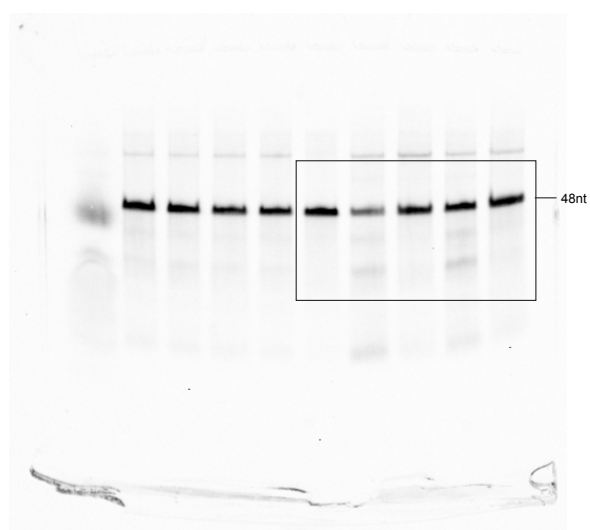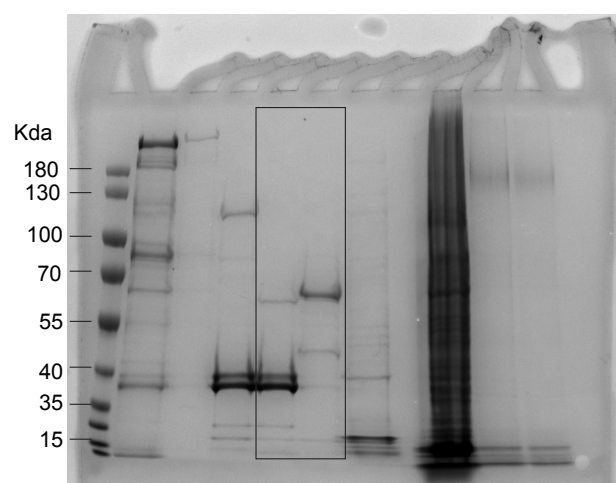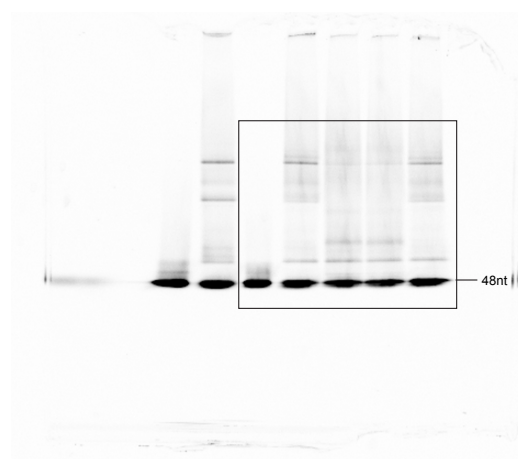

Supplement: Source Data Extended Data Fig. 10 — Unprocessed gels. [file 41594_2022_894_MOESM12_ESM.pdf]
